# Bond-selective photoacoustic imaging by converting molecular vibration into acoustic waves

**DOI:** 10.1016/j.pacs.2016.01.002

**Published:** 2016-02-01

**Authors:** Jie Hui, Rui Li, Evan H. Phillips, Craig J. Goergen, Michael Sturek, Ji-Xin Cheng

**Affiliations:** a Department of Physics and Astronomy, Purdue University, West Lafayette, IN 47907, USA; b Weldon School of Biomedical Engineering, Purdue University, West Lafayette, IN 47907, USA; c Department of Cellular and Integrative Physiology, Indiana University School of Medicine, Indianapolis, IN 46202, USA; d Department of Chemistry, Purdue University, West Lafayette, IN 47907, USA; e Purdue Institute of Inflammation, Immunology and Infectious Diseases, West Lafayette, IN 47907, USA

**Keywords:** Overtone absorption, Photoacoustic microscopy, Photoacoustic tomography, Intravascular photoacoustic, Lipid, Atherosclerosis, Tumor margin

## Abstract

The quantized vibration of chemical bonds provides a way of detecting specific molecules in a complex tissue environment. Unlike pure optical methods, for which imaging depth is limited to a few hundred micrometers by significant optical scattering, photoacoustic detection of vibrational absorption breaks through the optical diffusion limit by taking advantage of diffused photons and weak acoustic scattering. Key features of this method include both high scalability of imaging depth from a few millimeters to a few centimeters and chemical bond selectivity as a novel contrast mechanism for photoacoustic imaging. Its biomedical applications spans detection of white matter loss and regeneration, assessment of breast tumor margins, and diagnosis of vulnerable atherosclerotic plaques. This review provides an overview of the recent advances made in vibration-based photoacoustic imaging and various biomedical applications enabled by this new technology.

## 1 Introduction

Molecular vibration is the basis of numerous microscopy approaches and enables the detection of specific molecules within cells and tissues. These approaches include Raman scattering, infrared absorption, and near-infrared (NIR) absorption, which have been widely used for chemical imaging in biomedicine [1], [2], [3]. Similarly, nonlinear vibrational methods, such as coherent anti-Stokes Raman scattering [4], [5] and stimulated Raman scattering [6] microscopies, have enabled new discoveries in biology [7] on account of their high sensitivity and 3D spatial resolution. However, all these approaches have limited imaging depth on the order of a few hundred micrometers due to significant optical scattering in biological tissue. Thus, their potential applications at the organ level *in vivo* and in clinical settings are restricted.

A deep-tissue imaging modality able to maintain both high chemical selectivity and spatial resolution would certainly satisfy the functional requirements for many diagnostic applications in biomedicine. A promising approach is the development of photoacoustic (PA) imaging platforms, which combine optical excitation with acoustic detection. With this approach, the imaging depth is significantly improved, as acoustic scattering by biological tissue (∼1.2 × 10^−3^ mm^−1^ in human skin at 5 MHz) [8] is more than three orders of magnitude weaker than optical scattering (∼10 mm^−1^ in human skin at 700 nm) [9]. Unlike nonlinear optical microscopy that relies on tightly focused ballistic photons, the diffused photons contribute equally to the generation of PA signal and thus further enhance the penetration depth. Over the past decade, researchers have developed various PA imaging platforms, including photoacoustic microscopy (PAM) [10], [11], photoacoustic tomography (PAT) [10], [12], [13], photoacoustic endoscopy (PAE) [14], [15], and intravascular photoacoustic (IVPA) imaging [16]. Many excellent review articles provide comprehensive insight into different aspects of the imaging technology [17], [18], [19], applicable contrast agents [20], [21], [22], and a variety of biomedical applications [23], [24], [25], [26]. In most of the aforementioned applications, the PA signal comes from the electronic absorption of endogenous tissue pigments, such as hemoglobin and melanin, or from exogenous contrast agents, such as nanoparticles and dyes.

Molecular vibrational transitions in biological tissue have recently been demonstrated as a novel contrast mechanism for PA imaging. It describes the periodic motion of atoms in a molecule with typical frequencies ranging from 10^12^ to 10^14^ Hz. The molecular population in the *i*th vibrationally excited state relative to the ground state follows the Boltzmann’s distribution law as *N*_i_*/N*_0_ *=* exp(−Δ*E*/*kT*), where Δ*E* is the energy gap, *T* is the temperature, and *k* is the Boltzmann constant. Thus, the Boltzmann distribution describes how the thermal energy is stored in molecules. When the incident photon energy matches the transition frequency between the ground state and a vibrationally excited state, the molecule absorbs the photon and jumps to the excited state. During subsequent relaxation of the excited molecule to the ground state, the thermal energy is converted into acoustic waves detectable by an ultrasound transducer.

The fundamental vibrational transitions in the mid-infrared wavelength region have been previously exploited for PA detection of glucose in tissues [27]. Nevertheless, this approach is limited in detecting molecules only tens of micrometers under the skin, where strong water absorption in the mid-infrared region predominates. Vibrational absorption with minimal water absorption can occur in two ways. One is through the stimulated Raman process and the other is through overtone transition. In stimulated Raman scattering, the energy difference between the visible or NIR pump and Stokes fields is transferred to the molecule to induce a fundamental vibrational transition. The concept of stimulated Raman-based PA imaging has been previously demonstrated [28], [29]. However, because stimulated Raman scattering is a nonlinear optical process relying on ballistic photons under a tight focusing condition, this approach is not suitable for deep-tissue imaging. The overtone transition is based on the anharmonicity of chemical bond vibrations. Taking the C—H bond as an example, the first, second, and third overtone transitions occur at around 1.7 μm, 1.2 μm, and 920 nm, respectively, where water absorption is locally minimized. Since C—H bonds are one of the most abundant chemical bonds in biological molecules including lipids and proteins, photoacoustic detection of C—H bond overtone absorption offers an elegant platform for mapping the chemical content of tissue with penetration depths up to a few centimeters.

In the following sections, we introduce the mechanism for vibration-based PA signal generation. Then, applications of vibration-based PA imaging in forms of microscopy, tomography, and intravascular catheter will be reviewed, followed by a discussion of the improvements needed to overcome technical challenges that limit translation of these imaging modalities to the clinic.

## 2 Vibrational absorption as a photoacoustic contrast mechanism

### 2.1 Photoacoustic signal generation based on molecular overtone absorption

**Fig. 1:**
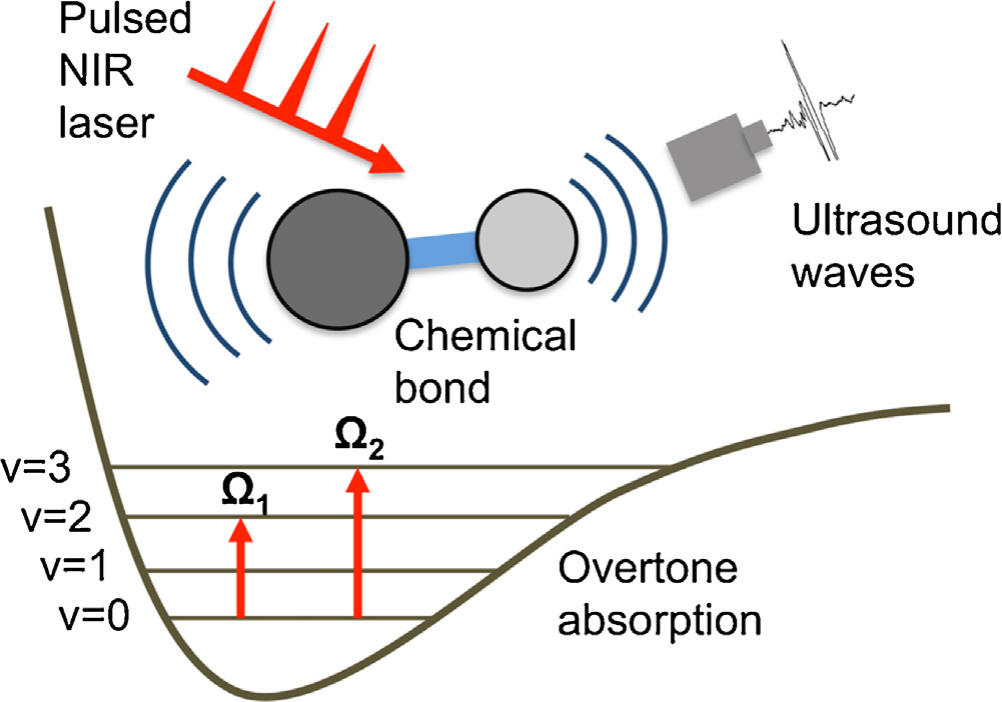
Schematic of vibration-based PA signal generation and the 1st and 2nd overtone absorption of a molecule. *v* denotes the vibrational energy level; NIR demotes near infrared.

Vibration-based PA signals arise from the molecular overtone transitions and combinational band absorptions, which are allowed by anharmonicity of chemical bond vibration. According to the anharmonicity theory, the transition frequency for an overtone band has the following relation with the fundamental frequency, Ω*_n_* *=* Ω_0_*n*-*χ*Ω_0_(*n* + *n*^2^), where Ω_0_ is the transition frequency of fundamental vibration, *χ* is the anharmonicity constant, and *n* = 2,3… representing the first, second, and subsequent overtones. When the frequency of an incident pulsed laser matches the transition frequency of an overtone, the energy of the incident photons is absorbed and then induces a local rise in temperature. When both thermal and stress confinements are satisfied [30], the accumulated heat is subsequently released through a thermal-elastic expansion in tissue, which generates acoustic waves detectable by an ultrasound transducer. Fig. 1 depicts this process for PA signal generation based on first and second overtone transitions. The generated signal contains depth-resolved information of absorbers on which the image reconstruction is grounded. Compared to diffuse optical tomography, the integration of NIR spectroscopy with ultrasound detection eliminates the scattering background.

Through conversion of molecular vibration into acoustic waves, vibration-based PA imaging enables the visualization of different molecules and chemical components in biological tissue. Thus far, CH_2_-rich lipids [31], [32], [33], [34], [35], [36], CH_3_-rich collagen [33], O—H bond-rich water [37], nerve [38], [39], intramuscular fat [34], [40], and neural white matter [41] have all been investigated. Particularly, the detection of overtone absorption of C—H bonds has recently drawn attention [42], [43], [44], [45], [46], [47], [48], since C—H bonds are highly concentrated in certain types of biological components, such as lipid and collagen. The presence of these molecules or components is directly related to several clinically relevant diseases, including atherosclerosis and cancers.

### 2.2 New optical windows for photoacoustic imaging

**Fig. 2:**
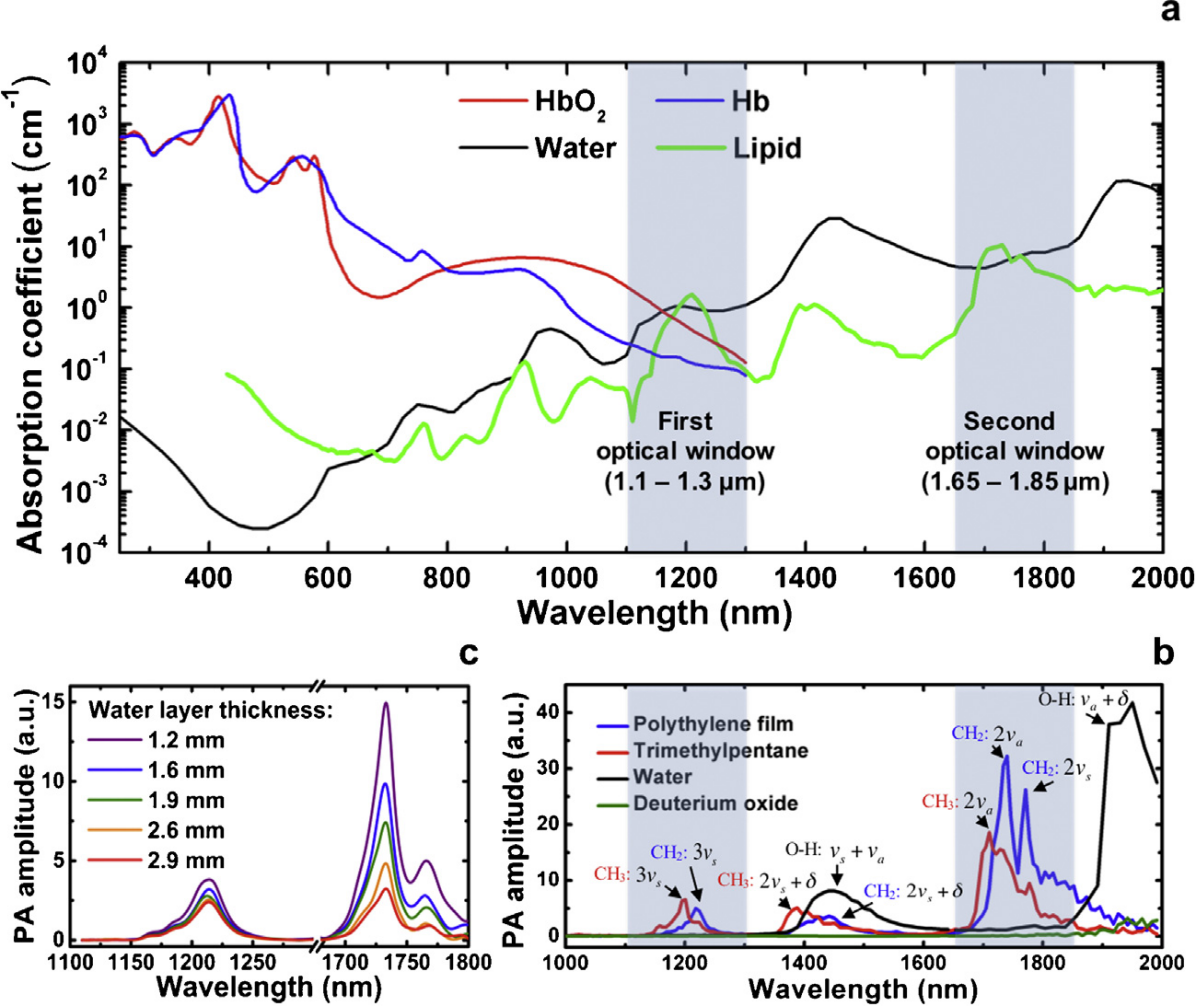
Two spectral windows for vibration-based PA imaging. (a) Optical absorption spectra of water (from Ref. [53]), lipid (from Refs. [54], [55]), oxygenated (HbO_2_) and deoxygenated (Hb) (from Ref. [50]) showing that the first optical window lies between 1.1 and 1.3 μm and the second window lies between 1.65 and 1.85 μm. (b) Vibration-based PA spectra of different chemical bonds or groups with absorption band assignments. *ν*_s_ and *ν*_a_ denote symmetric stretching and anti-symmetric stretching of chemical bond, respectively. (b) Vibration-based PA spectra of a C—H bond-rich sample (polyethylene film) with a varying water layer thickness. Adapted with permission from Ref. [34] (b, c).

Using vibrational absorption, researchers have conducted PA spectroscopic studies of various molecules in biological specimens [31], [34], [35], [49]. These efforts were aimed at identifying suitable spectral windows to visualize different biological components, as well as differentiate them based on their vibrational spectral signatures. As shown in Fig. 2a, two new optical windows have been identified for bond-selective photoacoustic imaging (highlighted in blue between 1100–1300 nm and 1650–1850 nm), where the absorption coefficient of C—H bond-rich specimens is maximized and water absorption is locally minimized. The electronic absorption of hemoglobin [50] is dominant in the visible to NIR wavelength range (*i.e.*, 400 nm to 1.1 μm) and it overwhelms the third- and higher-order C—H overtone transitions in the same range. For longer wavelengths in the range of 1.1–2.0 μm, the optical absorption from hemoglobin has been significantly reduced. In particular, in the first optical window, the hemoglobin absorption [50] is close to one order of magnitude smaller than lipid absorption. The whole blood in the second optical window (1650–1850 nm) exhibits almost the same spectrum as pure water [50], the major content of blood [51], [52], [53]. Although the absorption coefficient of lipid [54], [55] is 1–2 time larger than that of water in both optical windows, the fat constituent in tissue provides much higher contrast than water in vibration-based PA imaging. This observed phenomenon in PA imaging experiments can be explained by the following theoretical prediction and quantitative analysis.

Theoretically, the initial PA signal amplitude is described by *p*_0_ = *ξΓμ*_a_*F*, where *ξ* is a constant related to the imaging system, *Γ* is the Gruneisen parameter of tissue, *μ*_a_ is the absorption coefficient of tissue, and *F* is the local light fluence. The Gruneisen parameter can be further expressed as *Γ=βν*_s_^2^/*C*_p_, where *β* is the isobaric volume expansion coefficient, *ν*_s_ is the acoustic speed, and *C*_p_ is the specific heat. In the equation, only *Γ* and *μ*_a_ are dependent on absorbers in tissue. Thus, the vibration-based PA contrast of fat versus water can be expressed as *p*_0_fat_/*p*_0_water_ = (*μ*_a_*Γ*)_fat_/(*μ*_a_*Γ*)_water_. Based on the Gruneisen parameter and absorption coefficient of fat and water listed in Table 1 [9], [54], [56], the PA contrast of fat versus water is 9.6–12.4 and 10.9–14.0 at 1210 and 1730 nm, respectively. These parameters make vibration-based PA imaging a valid platform for selective mapping of fat or lipids in a complex tissue environment. Based on the same parameters, the fat signal amplitude at 1730 nm is 6.4 time of that at 1210 nm, largely due to the stronger absorption of lipid at 1730 nm.

Detailed analysis of the PA spectra of C—H, O—H, and O—D bonds further verified these two optical windows [57]. Fig. 2b shows the PA spectra of polyethylene film, trimethylpentane, water, and deuterium oxide. These spectra have contributions from the absorption profiles of methylene groups (CH_2_), methyl groups (CH_3_), O—H, and O—D bonds, respectively. According to the spectrum of polyethylene film, the peak at ∼1210 nm comes from the second overtone transition of the symmetric stretching of CH_2_ [58]. The broad peak located from 1350 to 1500 nm is attributed to the combinational band of symmetric stretching and bending of CH_2_. The two primary peaks at ∼1.7 μm are thought to be the first overtone of CH_2_ [58], which are caused by the anti-symmetric stretching and symmetric stretching, respectively [58]. For trimethylpentane, the 1195 nm peak corresponds to the second overtone transition of CH_3_ symmetric stretching [58]. The combinational band has a main peak at ∼1380 nm [58]. The primary peak at ∼1700 nm is thought to be the first overtone of anti-symmetric stretching of CH_3_ [58]. Although O—H bonds have combinational bands at ∼1450 and ∼1950 nm, respectively, its absorption is locally minimal in the first and second overtone windows of C—H bonds. Due to the heavier mass of deuterium, the prominent overtone and combinational bands of D_2_O have their corresponding peaks at longer wavelengths. Thus, it has been widely used as an acoustic coupling medium for vibration-based PA imaging [34], [57].

As shown in Fig. 2c, a PA spectroscopic study of polyethylene film with a varying water layer thickness suggests that the second overtone of C—H bonds is peaked at ∼1.2 μm, while the first overtone corresponds to the peak at ∼1.7 μm [34]. Compared with 1.2 μm excitation, 1.7 μm excitation produces a ∼6.3 times stronger PA signal in the absence of water [34], which is consistent with aforementioned theoretical calculation. The signal amplitude drops with the thickness of the water layer and has the same level as 1.2 μm excitation when the water layer thickness reaches 3–4 mm. Thus, a 1.7 μm wavelength is favorable for intravascular photoacoustic imaging considering the relatively large absorption coefficient of the first overtone and the diminished optical scattering caused by blood at longer wavelengths [36], [47], [52], [57]. The second overtone however is suitable for a tomographic configuration that requires larger penetration depths due to smaller water absorption at 1.2 μm [59]. These spectral signatures were utilized for different biomedical applications, as reviewed below.

## 3 Applications through vibration-based photoacoustic microscopy

**Fig. 3:**
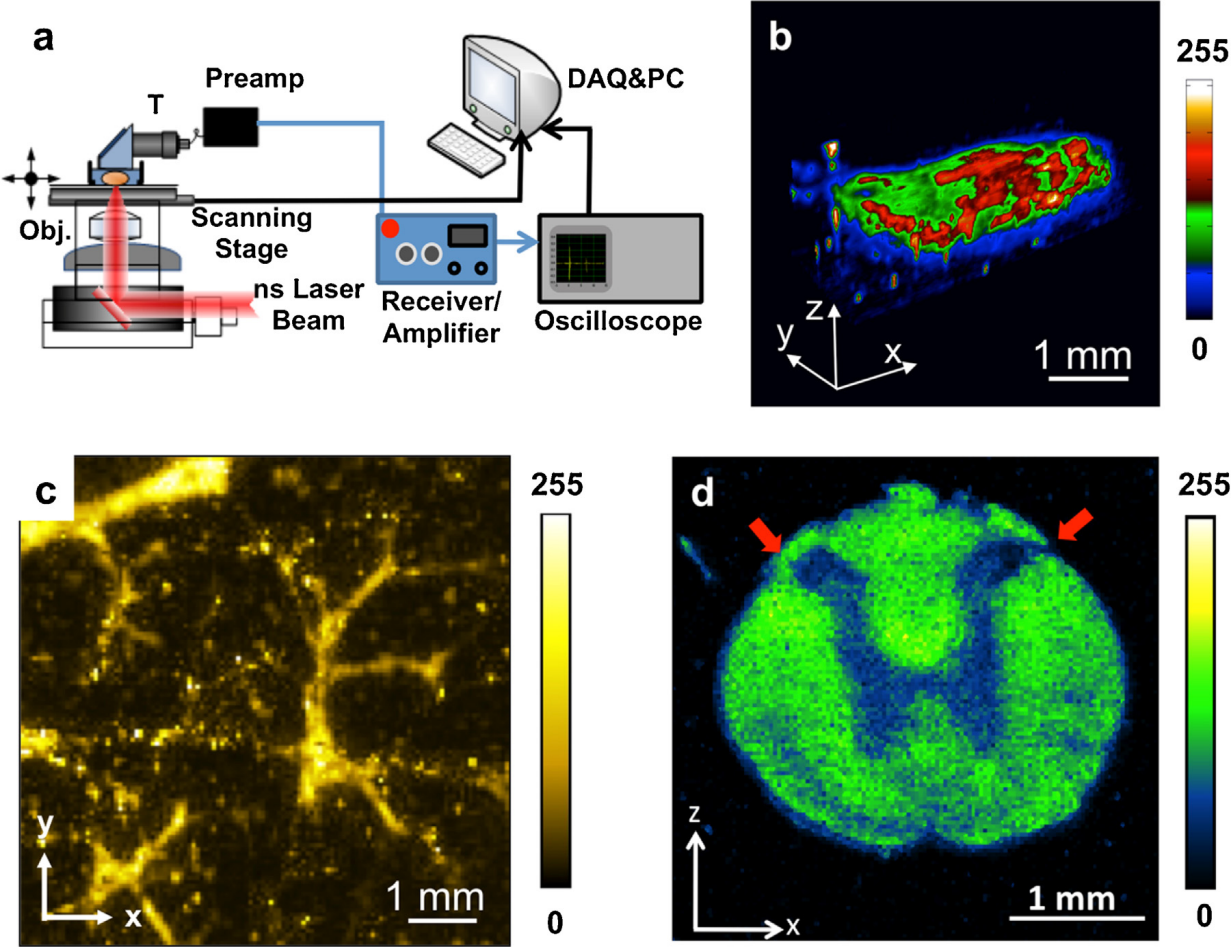
A PAM system and enabled representative applications enabled in the new optical windows. (a) Schematic of a typical PAM system. T, ultrasound transducer. (b) 3D image of lipid bodies in Drosophila 3-instar Larva at ∼1200 nm. (c) Image of intramuscular fat at 1197 nm performed with a Raman laser. (d) Image of white matter in a normal rat spinal cord at 1730 nm showing the contrast difference between white matter and gray matter. Red arrows indicate the dorsolateral surface of the cord above dorsal horn. Adapted with permission from Ref. [31] (a, b), Ref. [40] (c), and Ref. [41] (d).

Based on its high spatial resolution, deep penetration depth, and rich optical absorption contrast, PAM has been used extensively and enabled new discoveries in biology and medicine. Using vibrational absorption, new applications are explored through PAM in the relevant optical windows. In a typical PAM setup, an inverted microscope is employed to direct the excitation light (Fig. 3a) which can be generated by Nd:YAG pumped optical parametric oscillator (OPO) [31], [38], [60] or a Raman laser [40]. An achromatic doublet lens or objective is applied to focus the laser light into a sample. A focused ultrasonic transducer records the time-resolved PA signal from the acoustic focal zone. According to the time of flight, each laser pulse can be used to generate an A-line. By raster scanning the sample in the *X*–*Y* direction, a three-dimensional image can be acquired.

### 3.1 Mapping lipid bodies in Drosophila 3rd-instar larva

One important application for PAM in the new optical windows is to map the lipid bodies in Drosophila 3rd-instar Larva. Drosophila melanogaster is one of the genetically best-known and widely used model organisms for genetic, behavioral, metabolic, and autophagic studies [61], [62], [63]. Since lipids have strong optical absorption due to the second overtone transition of the C—H bond, Wang et al. performed 3D imaging of lipid body of a whole 3rd-star larva *in vivo* (Fig. 3b). The imaging result shows that lipid storage is mainly distributed along the anterior-posterior and the ventral-dorsal axis. This demonstrated capability of label-free visualization of adipose tissues in Drosophila is important for the rapid determination of phenotype, which will decrease the time required to conduct genetic screens for targets of fat metabolism and autophagy in this model organism [64], [65].

### 3.2 Mapping intramuscular fat

Intramuscular lipids are associated with insulin resistance, which is related to a range of metabolic disorders including type 2 diabetes, obesity, and cardiovascular diseases [66], [67]. However, the assessment of intramuscular fat is difficult since current deep-tissue imaging modalities cannot provide chemical contrast. Li et al. reported the feasibility of performing intramuscular fat mapping with a Ba(NO_3_)_2_ crystal-based Raman laser [40]. The Raman laser provided an output with wavelength of 1197 nm. The signal from fat at 1197 nm is strong and the contrast nearly disappeared at 1064 nm, which indicates a strong absorption at 1197 nm due to the second overtone transition of C—H bond. The muscle sample was also imaged in three dimensions with an imaging depth of 3 mm, where the fat structure was clearly reflected (Fig. 3c). This result shows the promise of using this technique for quantitative measurement of intramuscular fat accumulation in metabolic disorders.

### 3.3 Mapping white matter loss and regeneration

Each year, approximately 12,000 new cases of spinal cord injury are diagnosed in the U.S., causing tetraplegia or paraplegia. White matter loss is thought to be a critical event after spinal cord injury. Traditionally, such degeneration is measured by histological and histochemical approaches [68]. However, real-time imaging is not feasible and artifacts are often introduced during histological processing. Wu et al. used PAM with 1730 nm excitation to assess white matter loss after a contusive spinal cord injury in adult rats [41]. Owing to the abundance of CH_2_ groups in the myelin sheath, white matter in the spinal cord can be easily visualized (Fig. 3d). From the cross-sectional image, contrast from white matter is ∼2.5 times higher compared with grey matter. The absorption difference can be used to examine the morphology of white matter and changes in injured spinal cords. This study suggests that PAM based on first overtone transition of C—H bond could be potentially used to assess white matter loss during spinal cord injury and repair.

## 4 Applications through vibration-based photoacoustic tomography

**Fig. 4:**
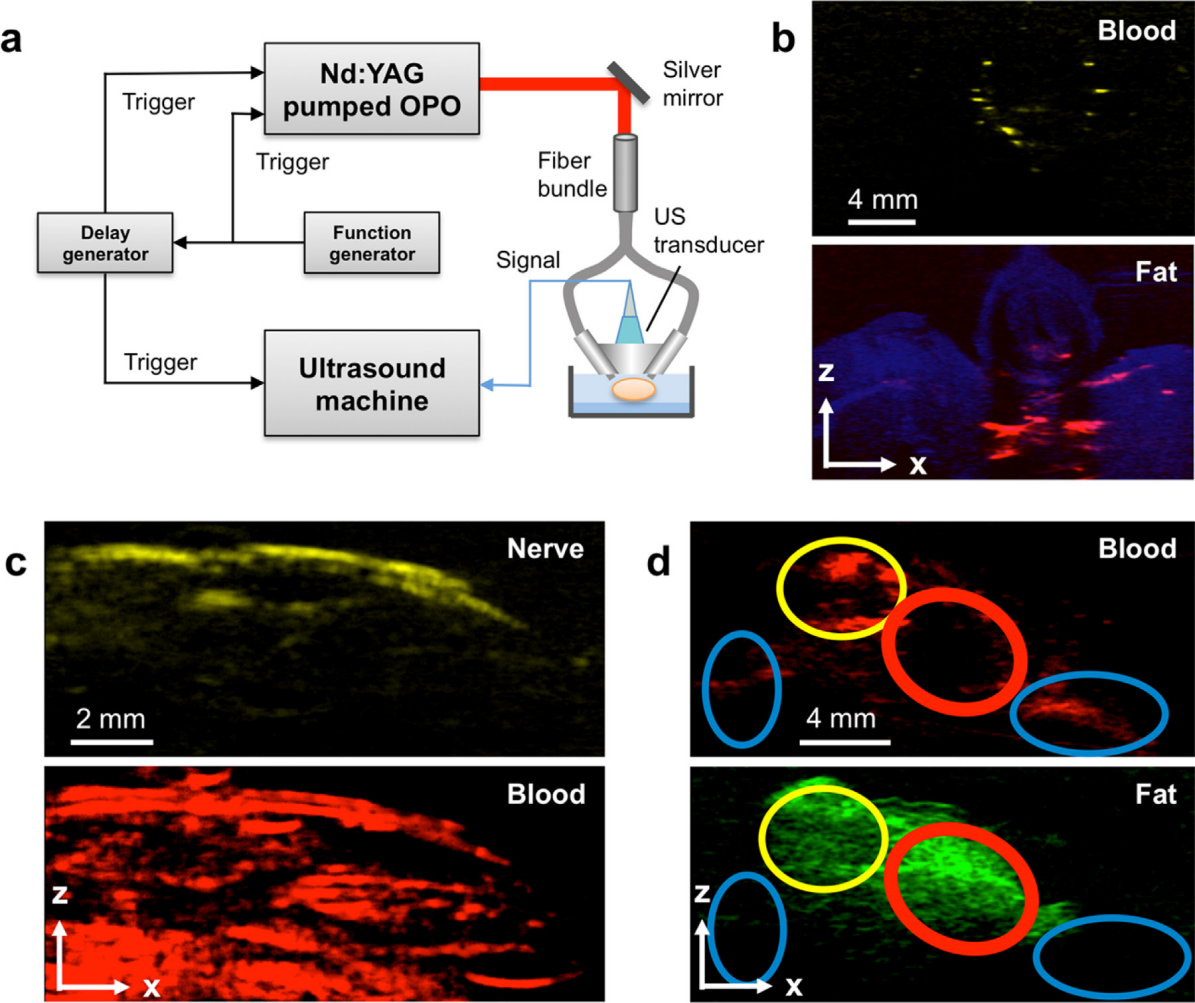
A PAT system and enabled representative applications enabled in the new optical windows. (a) Schematic of a typical PAT system. OPO, optical parametrical oscillator; US, ultrasound. (b) Images of modeled atherosclerotic carotid artery with contrast from lipid and blood. (c) Images of mouse peripheral nerve with contrast from fat and blood. (d) Image of breast tumor margin with contrast from fat and blood. Red oval indicates a normal tissue area with fat and scattered fibrous tissue; yellow oval indicates angiogenesis and invasive tumor with scattered fat tissue; blue oval indicates tumor with dense fibrous tissue. Adapted with permission from Ref. [90] (a, d), Ref. [78] (b), and Ref. [39] (c).

By taking advantage of signal generation from diffused photons, PAT penetrates deeper than PAM and expands the imaging scale from the cell and tissue to whole organ level [13]. The high scalability of PAT is achieved through a trade-off in spatial resolution for improved imaging depth. Moreover, the imaging scale can vary with the specific needs of PAT applications. Current applications for PAT include lymphatic [69] and sentinel lymph node [70], [71] mapping, superficial [72] and deep [73] vessel mapping, and tumor imaging [74], [75]. The key advantages of this technique are noninvasiveness, superior depth penetration, and chemical-selectivity without the need for exogenous agents. For superior penetration depth, the experimental set-up requires integration of a high power laser with a low-frequency ultrasound array. Fig. 4a shows a typical PAT system [39]. Briefly, a customized OPO laser (NT352C, EKSPLA) generating a 10 Hz, 5 ns pulse train with wavelength tunable from 670 to 2300 nm was used as the light excitation source. An optical fiber bundle delivers the light to tissue through two rectangular distal terminals adjacent to an arrayed ultrasound transducer with center frequency of 21 MHz (MS250, FUJIFILM VisualSonics). The generated PA signal is then acquired and reconstructed as two-dimensional or three-dimensional tomographic images using the ultrasound system. Below we describe a range of applications using molecular overtone absorption for tomographic imaging of lipid-associated diseases.

### 4.1 Imaging a model of carotid atherosclerosis

Carotid artery atherosclerosis is a common underlying cause of ischemic stroke [76], [77]. Noninvasive imaging and quantification of the compositional changes within the arterial wall is essential for disease diagnosis. Current imaging modalities are limited by the lack of compositional contrast, inability to detect of non-flow-limiting lesions, and inadequate accessibility to patients (like magnetic resonance imaging). However, modified multispectral PAT has great potential for serving as a point-of-care device for early diagnosis of carotid artery disease in the clinic. Hui et al. tested this system to image *ex vivo* atherosclerotic human femoral arteries and tissue-mimicking phantoms [78]. We placed a 45-degree polished fiber-optic probe and a 21 MHz linear array transducer with 256 elements on opposite sides of the sample with a thick piece of chicken breast in order to mimic the *in vivo* conditions of carotid artery imaging through transesophageal excitation and external acoustic detection. Chemical maps of the blood and lipid in the lipid-laden vessel and fatty chicken breast were generated as shown in Fig. 4b.

Furthermore, for the tissue-mimicking phantom experiment, a piece of chicken breast was added between the excitation source and a polyethylene tube in order to analyze the signal-to-noise ratio and imaging depth in this set-up. An imaging depth of about 2 cm was achievable in this scenario while retaining chemical selectivity around 1210 nm and spectral discrimination (<10 nm) between the intramuscular fat in the chicken breast and the polyethylene tube. These results collectively show that this prototype fiber-optic probe design enables deep-tissue multispectral imaging and has translational potential as a minimally invasive diagnostic tool.

### 4.2 Imaging peripheral nerves

In a surgical procedure, iatrogenic damage to peripheral nerves is a leading cause of morbidity [79], [80], [81]. The resultant complications include temporary numbness, loss of sensation, and peripheral neuropathy [80], [82]. The accurate noninvasive visualization of nerve tissues relative to adjacent structures is of vital importance yet remains technically challenging. As myelin sheaths surrounding axons are abundant in lipids, there is an opportunity to apply PA imaging technique to discriminate nerves from adjacent tissues using lipid and blood as two different contrasts.

A preliminary feasibility study of nerve imaging was performed in a PAM configuration [38]. Clinical translation of this technique however is impeded by millimeter-scale imaging depth and slow imaging speed. Li et al. recently demonstrated the label-free *in vivo* tomographic visualization of mouse nerves through PAT based on second overtone absorption of C—H bonds with an imaging depth of at least 2 mm [39]. Spectroscopic imaging was performed in the optical window of 1100 to 1250 nm to discriminate lipid (∼1210 nm) from blood (<1150 nm). An algorithm called multivariate curve resolution alternating least squares (MCR-ALS) [83] was then applied to the spectroscopic image stack to resolve chemical maps from lipid and blood. As shown in Fig. 4c, the femoral nerve fiber was clearly resolved and distinguished from the adjacent femoral artery. Although this application does not require a greater imaging depth, it demonstrates chemical selectivity (using only two excitation wavelengths) and sufficient spatial resolution to discriminate adjacent structures with a large imaging field of view. It has the potential for label-free imaging of peripheral nerves in patients undergoing surgery.

### 4.3 Assessing breast tumor margins

Breast-conserving surgery, or lumpectomy, is a common procedure for breast cancer treatment [84], [85]. To prevent local cancer recurrence after lumpectomy, histology is performed to check whether the excised tumor specimen is surrounded by a sufficient amount of normal tissue [86], [87]. A re-operation is needed if a positive margin is identified. Currently, the re-operation rate ranges from 20% to 70% [88], [89]. This high re-operation rate highlights a pressing need for the development of an intraoperative device that is rapid, sensitive, label-free, and able to scan the entire tissue surface for accurate breast cancer margin assessment.

Recently developed multispectral PAT combining lipid with blood contrast provides a compelling opportunity to meet this need [90]. Specific to breast cancer, multispectral PAT, as shown in Fig. 4a, was applied for margin detection in the optical window from 1100 to 1250 nm. In this window, the distribution of fat and blood were visualized after acquisition of a multispectral image stack. The image stack was processed through the MCR-ALS algorithm, generating chemical maps for those two major components (Fig. 4d). Based on the imaging results and a comparison with histology results, the area with fat and lacking hemoglobin contrast was assigned to be normal tissue with fat and scattered fibrous tissue (red oval). The area with hemoglobin contrast and fat indicated angiogenesis and invasive tumor with scattered fat tissue (yellow oval). The area without fat contrast indicated tumor tissue with dense fibrous tissue (blue oval). These results collectively demonstrated the capacity of tumor margin assessment based on the contrast of hemoglobin and fat. This imaging configuration maintains an imaging depth of up to 3 mm, which is sufficient for determining breast tumor margins (typically 1–2 mm of tumor-free tissue are necessary for a margin to be negative). With 100% sensitivity, the system can successfully detect breast tumor margin and opens a new way for clinical intraoperative breast tumor margin assessment. A similar approach using blood and lipid as two complementary contrasts has recently been demonstrated to visualize the vasculature and external boundaries of healthy lymph nodes across their depth (<6 mm) [91]. Future studies will be useful for “indirect detection” of cancerous nodes in which the structure and composition are expected to change.

## 5 Intravascular photoacoustic imaging of lipid-laden plaques

Beyond microscopic studies of lipid-laden plaques inside an atherosclerotic artery, IVPA has been intensively investigated over the past few years. As is widely known, cardiovascular disease is the number one cause of death in the United States. The majority of acute fatal incidents with cardiovascular disease are due to vulnerable plaques, which are at a high risk for rupture and thrombosis [92], [93]. Pathophysiological findings suggest that these vulnerable lesions contain a large lipid core covered by a thin fibrous cap and are located in areas of high shear stress within the coronary arterial wall [94], [95]. Current imaging modalities either lack compositional contrast or sufficient imaging depth for this application. Furthermore, no existing imaging tools can reliably and accurately diagnose vulnerable plaques in live patients [96]. However, IVPA maintains high resolution, chemical selectivity, and sufficient imaging depth to characterize vulnerable plaques. It has great potential to be developed as a life-saving device for diagnosis of vulnerable plaques.

### 5.1 Imaging lipid-laden atherosclerotic plaques

The current translational goal for IVPA imaging is to detect lipid-laden plaques with high accuracy and specificity. Thus, the selection of an optimal wavelength to excite lipids becomes the primary objective. PA imaging of lipid-rich plaques has been demonstrated using different wavelengths [35], [97], [98]. However, the PA signal from lipids between 400 and 1100 nm is greatly overwhelmed by hemoglobin absorption, and is not suitable for *in vivo* applications. When the wavelength exceeds 1.1 μm, hemoglobin absorption is minimal, but significant water absorption due to vibrational transition of O—H bonds attenuates light intensity inside the biological tissue. Nevertheless, two optical windows have been revealed for PA detection of overtone absorption of C—H bonds, where lipids can be imaged at ∼1.2 μm and ∼1.7 μm as discussed before [31], [36], [44].

**Fig. 5:**
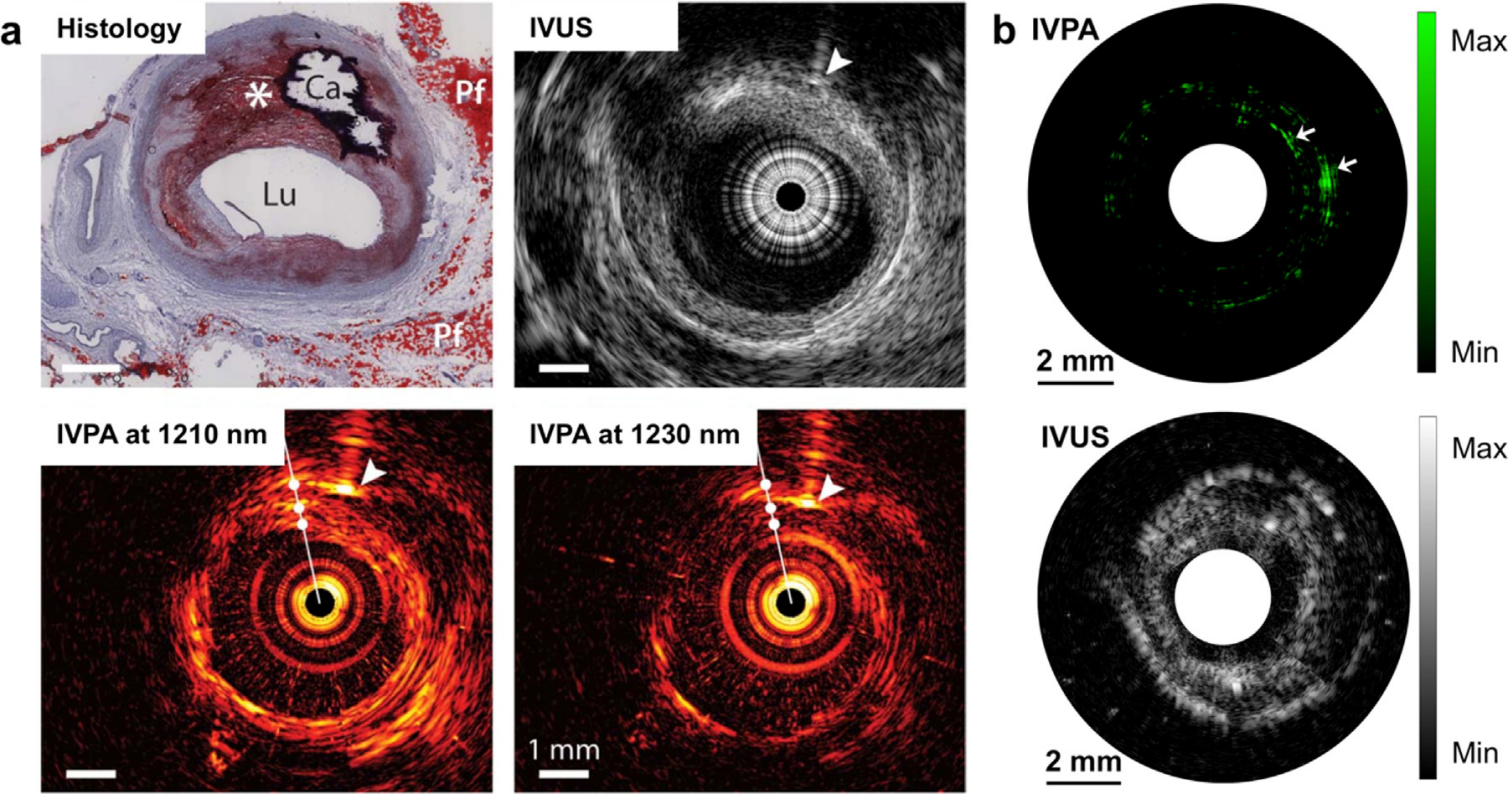
IVPA/IVUS imaging of lipid-laden atherosclerotic plaque. (a) IVPA/IVUS imaging of an advanced human atherosclerotic plaque in 1.2 μm optical window: histology with Oil Red O stain; IVUS image; IVPA image at 1210 nm (high lipid absorption); IVPA image at 1230 nm (low lipid absorption). *, lipid-rich plaque; Ca, calcified area; arrowheads, a needle used for marking. (b) IVPA/IVUS imaging of an excised human femoral artery in 1.7 μm optical window: IVPA image at 1724 nm with white arrows indicating large lipid deposition; IVUS image. Adapted with permission from Ref. [32] (a) and Ref. [99] (b).

Grounded on these two lipid-specific optical windows, a series of IVPA imaging developments has been reported. Jansen et al. demonstrated the first IVPA imaging result of human artery with 1210 nm excitation of the second overtone of C—H bond [32]. As seen in Fig. 5a, the histology shows a large eccentric lipid-rich lesion, as well as a calcified area and regions of peri-adventitial fat, which is confirmed by IVUS image. The IVPA image at 1210 nm exhibits a bright signal along the intimal border, and also from deeper tissue layers in the eccentric plaque and the peri-adventitial fat in the bottom right corner, compared with IVPA image at 1230 nm. Wang et al. tested the feasibility of IVPA imaging of atherosclerotic rabbit aorta at 1.7 μm in the presence of luminal blood [36]. A preliminary study of *in vivo* IVPA imaging was also performed at 1720 nm in a rabbit model [43]. These results suggest that *in vivo* IVPA imaging is possible even without flushing luminal blood with saline, a necessary step for imaging coronary arteries with optical coherence tomography. Recently, with the availability of high-repetition-rate laser sources, a few research groups have developed high-speed IVPA imaging at ∼1.7 μm [99], [100], [101]. Hui et al. demonstrated the high-speed IVPA imaging of human femoral artery *ex vivo* at 1 frame per sec as shown in Fig. 5b [99]. The IVPA and IVUS images of the atherosclerotic artery reveal complementary information in the artery wall. The lipid deposition in the arterial wall indicated by white arrows at 2 and 3 o'clock directions, which is not seen in the IVUS image, shows clear contrast in the IVPA image.

### 5.2 High-speed laser sources

**Fig. 6:**
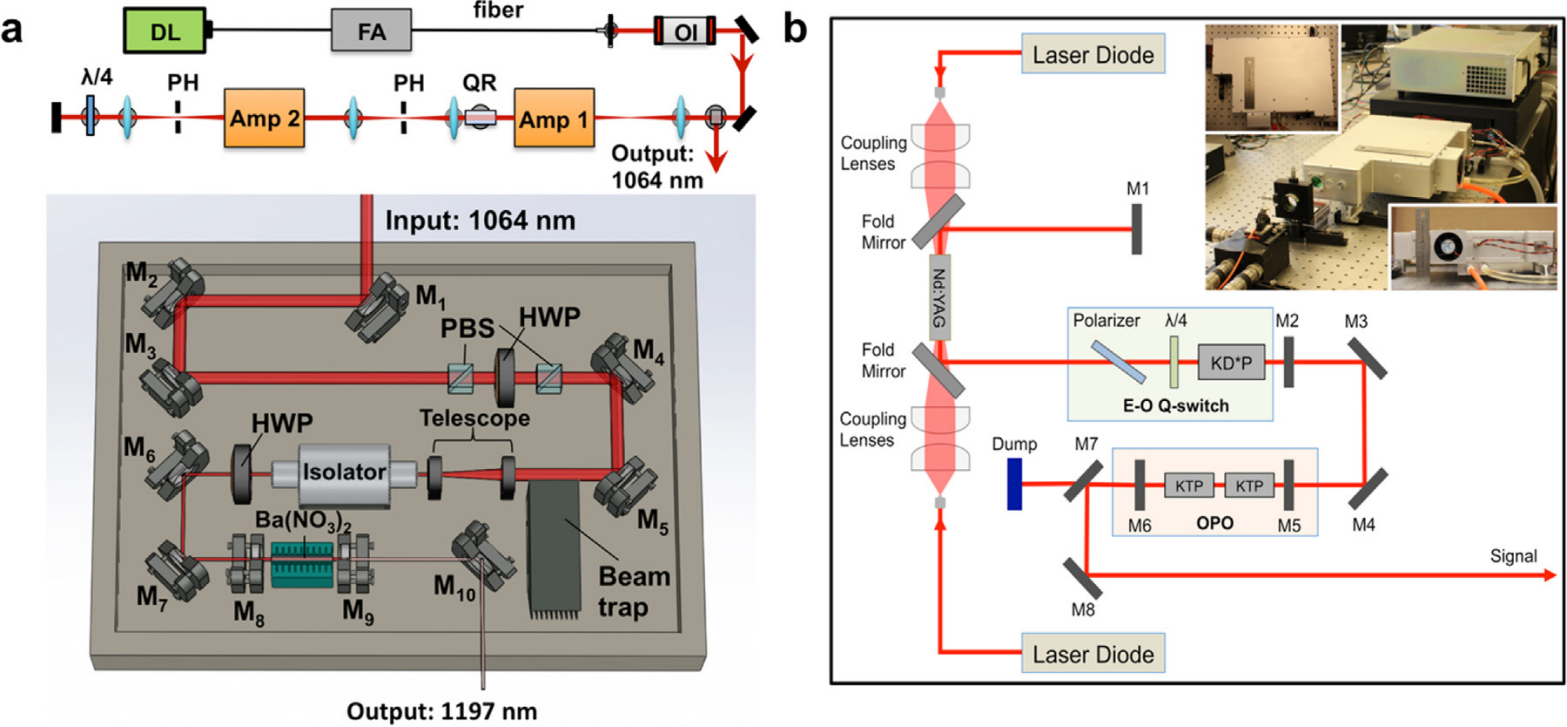
High-repetition-rate laser sources developed for IVPA imaging. (a) Schematics of 2 kHz MOPA-pumped Raman laser with an output of 1197 nm. Amp: amplifier; PH: pin hole; QR: quartz rotator; OI: optical isolator; FA: fiber amplifier; DL: Directly modulated diode laser; M_1_–M_7_: 45-degree 1064 nm reflective mirror; PBS: polarizing beam splitter; HWP: half wave plate; M_8_: resonator end mirror; M_9_: output coupler; M_10_: silver mirror. (b) Schematics of 500 Hz KTP-based OPO laser with an output of 1724 nm. M_1_, M_2_, M_5_, M_6_, flat mirrors; M_3_, M_4_, M_8_, reflective mirrors; M_7_, dichroic mirror; KD*P, potassium dideuterium phosphate Pockels cell; KTP, potassium titanyl phosphate. The inset shows the pictures of the actual laser system. Adapted with permission from Ref. [47] (a) and Ref. [99] (b).

IVPA imaging has been widely considered a promising technique for the diagnosis of vulnerable plaque in the arterial wall of live patients. However, the translation of the imaging technology from bench to bedside has been stifled by its slow imaging speed, mainly due to lack of suitable laser sources to excite the molecular overtone transitions at a high repetition rate. Wang et al. recently demonstrated a 2-kHz master oscillator power amplifier (MOPA)-pumped barium nitrite (Ba(NO_3_)_2_) Raman laser, which enabled the high-speed IVPA imaging [47]. In the laser system, a 1064 nm pulsed laser at a repetition rate of 2 kHz generated by the MOPA laser was used to pump the Ba(NO_3_)_2_-based Raman shifter (Fig. 6a). Through a stimulated Raman scattering process, the 1064 nm pump laser is converted to an 1197 nm output, which matches the second overtone vibration of C—H bond and thus can be used to excite the lipid-rich plaques. The high-speed IVPA imaging with this laser was validated using the iliac artery from a pig model at a speed of 1 frame per sec, which is nearly two orders of magnitude faster than previously reported systems.

Since 1.7 μm optical window is more optimal for *in vivo* IVPA imaging when compared with 1.2 μm, the laser development having output at ∼1.7 μm with high pulse energy and high repetition rate is of great importance toward the *in vivo* applications of IVPA imaging. More recently, several research groups have developed such laser sources based on different technologies with a repetition rate of kHz levels and applied them to high-speed IVPA imaging [99], [100], [101]. As one example, shown in Fig. 6b, a potassium titanyl phosphate (KTP)-based OPO laser has an output at 1724 nm with pulse energy up to 2 mJ and with pulse repetition rate of 500 Hz [99]. In order to obtain high pulse energy at high repetition rate, two KTP crystals were cut with a special angle and placed with adverse orientation in the OPO, which effectively minimized the walk-off effect. This laser enabled imaging of human artery at a speed of 1 frame per sec with a cross-sectional IVPA image composed of 500 A-lines. This speed greatly reduces the ambiguity caused by slow imaging speed and can be further used for preclinical *in vivo* imaging. However, in order to make IVPA competitive in the clinic, the repetition rate needs to be further improved to the order of 10 kHz [99].

## 6 Discussion

Harnessing its high depth scalability and endogenous contrast mechanism, vibration-based PA imaging has opened up a range of biomedical applications in forms of microscopy, tomography, and intravascular imaging, as well as technical challenges for their translation to the clinic. PAM with overtone absorption of C—H bonds as the contrast can achieve ultrasonic resolution in deep tissue regime. As lipids are rich in CH_2_-groups, PAM offers opportunities for lipid imaging, which is often related to disease severity, including type 2 diabetes and white matter loss and regeneration. Using an OPO as the light exciting source, multispectral PAM can be achieved with spectral signatures of molecules. Currently, with ∼20 MHz ultrasound transducer, the lateral resolution of vibration-based PAM is ∼70 μm [31]. However, it potentially can be increased by 10 times with optical-resolution PAM configuration through a ∼75 MHz transducer and objective lens [102]. Because of light focusing and sample scanning scheme, the speed of PAM is limited, which hinders its translation from bench to bedside. However, imaging speed may be improved by translating the transducer instead of the sample stage [103].

PAT in the new optical windows offers new opportunities for its use in biomedicine, but also some particular engineering challenges. As demonstrated in the previous examples, the ability to perform either two-color imaging or multispectral tomography lends the technology to different applications. For instance, a two-color PAT system for *ex vivo* breast tumor margin assessment may be advantageous for a compact mobile system designed for a clinical setting, whereas multispectral tomography is helpful for analyzing spectral differences in more complex tissue samples. The most salient advantage and future direction for vibration-based PAT though is the enabling of molecular-specific deep-tissue imaging applications. Vessels, nerves, and organs that accumulate pathological levels of lipid are of primary interest in this regard. Currently, magnetic resonance imaging, X-ray computed tomography, and ultrasound are used in the clinic to aid in the diagnosis and treatment monitoring of lesions, such as those in atherosclerosis and fatty liver disease [104], [105]. While magnetic resonance imaging provides excellent soft tissue contrast and adequate resolution for these applications, its cost and availability make it an impractical option in many cases. Ultrasound and PA imaging are well suited to clinical applications requiring functional and chemical-selective characterization while providing greater imaging depth than purely optical tomographic techniques. Furthermore, different configurations involving excitation or detection outside or within the body expand the capabilities and potential applications of vibration-based PAT.

Challenges for tomographic imaging, including vibration-based PAT, are present and require refinements in the engineering of these systems. Two significant challenges for *in vivo* imaging are the presence of clutter, or unwanted superficial signal enhancement, and volumetric imaging without motion artifacts. The first is mainly contributed by the distance between the illumination source and the imaging surface (*i.e.*, the irradiation distance) [104]. The geometry of the illumination source and detector may also help to alleviate this effect, which degrades the signal to background ratio. Volumetric imaging provides significantly more morphological and compositional information of large and complex regions. With handheld two-dimensional array transducers, it is important that it is stabilized in order to collect three-dimensional images. Furthermore, gating techniques (*i.e.*, respiration and cardiac gating) can significantly reduce motion artifacts from breathing and vessel motion in small animals, which have high heart and respiration rates. The integration of gating into PAT will be critical for future experiments examining complex lesions and areas of the body where motion is significant.

IVPA imaging has become one of the hot topics in the field of biomedical imaging. So far, it has shown great potential for clinical applications and is undergoing rapid development. To translate the imaging tool from bench to bedside, several significant challenges need to be resolved. The first and most pressing challenge is to develop a high-pulse-energy (mJ level) laser with a lipid-specific wavelength at 1.7 μm and with a pulse repetition rate at 10 kHz level. The high pulse energy would be large enough, but also under the ANSI safety limits [106], to ensure effective PA signal generation even through a thin layer of luminal blood. The high repetition rate would enable IVPA imaging at high speeds (∼30 frames/sec), comparable with the speed of many IVUS systems. The second one is to design a clinically relevant IVPA catheter. The catheter should be further miniaturized to ∼1 mm or less in diameter for clinical practice, as well as maintain excellent detection sensitivity. Better sensitivity could help reduce the laser pulse energy, thus further reducing the challenge for development of a high-pulse-energy laser. In addition, a clinically relevant large animal used as a model for human atherosclerosis is also essential for the validation of IVPA imaging technology. With this model, the *in vivo* IVPA imaging procedure, the detection of lipid-laden plaques, and the clinical requirements of catheters in pressurized vessels and blood can be all tested and validated. Ultimately, IVPA imaging of living patients could have a phenomenal impact on both diagnosis and treatment of vulnerable plaques. Moreover, it has the potential to guide coronary stenting during percutaneous coronary intervention, as well as to stimulate the development of new cholesterol-lowering and anti-inflammatory therapeutics for atherosclerosis. Indeed, it has the potential to be a life-saving technology when used in clinical settings.

## Conflict of interest

The authors declare that there are no conflicts of interest.

## Figures and Tables

**Table 1:** Absorption coefficient and Gruneisen parameter of fat and water at 1.2 and 1.7 micron.

Tissue constituent	Tissue parameter	1210 nm	1730 nm	Ref.
Fat	*μ*_a_ (cm^−1^)	1.65	10.5	[54]
	*Γ*	0.7–0.9	0.7–0.9	[56]
Water	*μ*_a_ (cm^−1^)	1.00	5.63	[53]
	*Γ*	0.12	0.12	[9]
